# Experiments and Analysis of Close-Shot Identification of On-Branch Citrus Fruit with RealSense

**DOI:** 10.3390/s18051510

**Published:** 2018-05-11

**Authors:** Jizhan Liu, Yan Yuan, Yao Zhou, Xinxin Zhu, Tabinda Naz Syed

**Affiliations:** 1 Key Laboratory of Modern Agricultural Equipment and Technology, Ministry of Education, Jiangsu University, Jiangsu 212013, China; 2211616012@stmail.ujs.edu.cn (Y.Y.); 2211416008@stmail.ujs.edu.cn (X.Z.); 5102160315@stmail.ujs.edu.cn (T.N.S.); 2 College of Information Science and Technology, Nanjing Forestry University, Nanjing 210037, China; zy110301425@gmail.com

**Keywords:** citrus, RealSense, recognition, depth-sphere cut, algorithm

## Abstract

Fruit recognition based on depth information has been a hot topic due to its advantages. However, the present equipment and methods cannot meet the requirements of rapid and reliable recognition and location of fruits in close shot for robot harvesting. To solve this problem, we propose a recognition algorithm for citrus fruit based on RealSense. This method effectively utilizes depth-point cloud data in a close-shot range of 160 mm and different geometric features of the fruit and leaf to recognize fruits with a intersection curve cut by the depth-sphere. Experiments with close-shot recognition of six varieties of fruit under different conditions were carried out. The detection rates of little occlusion and adhesion were from 80–100%. However, severe occlusion and adhesion still have a great influence on the overall success rate of on-branch fruits recognition, the rate being 63.8%. The size of the fruit has a more noticeable impact on the success rate of detection. Moreover, due to close-shot near-infrared detection, there was no obvious difference in recognition between bright and dark conditions. The advantages of close-shot limited target detection with RealSense, fast foreground and background removal and the simplicity of the algorithm with high precision may contribute to high real-time vision-servo operations of harvesting robots.

## 1. Introduction

The rapid development of the global fruit and vegetable industry has contributed to agricultural upgrading and increased income for agricultural practitioners. According to the statistics of the Food and Agriculture Organization of the United Nations, global vegetable and fruit production increased by 3.7-times and 4.3-times, respectively, over the past 50 years [1]. However, the fruit and vegetable industry is labor-intensive. There are about 150 million people engaged in the daily planting and management of fruits and vegetables in China alone, for which harvesting takes up 40–50% of the total work [2,3].

With the advancement of science and technology, the production of field crops around the world has been fully mechanized. At the same time, mechanical equipment has been used in some fields, such as fruit and vegetable cultivation, field management, and so on. However, harvesting fresh fruits and vegetables still generally relies on people, which takes up most of the labor and is the most difficult area to utilize mechanized operations [2,4]. Therefore, robotic harvesting has been a hot topic in agricultural research. Although researchers have paid much attention to it, many challenges remain for efficient and reliable picking operations in the real agricultural environment. Until now, most fruit-harvesting robots have only been tested in laboratories. Therefore, the key problem, which could lead to a breakthrough for harvesting robots, is to promote the resolution, reliability and real-time applicability of fruit recognition and location.

Recognizing and locating fruits is a tough task for picking robots. Over the past few decades, charge-coupled device (CCD) cameras have been used to identify and locate fruit in most research works. However, CCD cameras are too light sensitive to provide reliable identification in natural scenes [5,6]. Real-time fruit recognition and location can be affected by some problems: too much redundant information, the significant amount of computing needed to identify fruit objects and the complexity of image matching of the objects’ locations. The method of fruit recognition based on a color image is often useless for problems of a similar color of fruits and leaves, the overlap of fruit portions, uneven fruit color and bright spots on images in a nonstructural environment. Various targeted studies have only achieved limited improvements of those problems and have not proposed a satisfactory solution for them [7,8,9]. In recent years, depth sensors have been used in fruit recognition and location, as they can obtain the object’s characteristic information, without having to rely on the color information completely. Low-cost consumer red-green-blue-depth (RGB-D) cameras, such as Microsoft Kinect and Intel RealSense [10,11], have revolutionized the field of fruit recognition and location technology because they can obtain color and three-dimensional (3D) depth information of an object in real time synchronously.

The existing recognition and location algorithms, which are based on RGB-D, are different for different information. Wang et al. [12] used Kinect to recognize on-tree mangoes by using the histogram of oriented gradients (HOG) and an ellipse-fitting algorithm based on a color image and estimated the size based on the depth information. García-L and Morales [13] used an RGB image acquired by Asus Xtion to find red spheres that emulate ripe tomatoes and used the depth information to locate the objects. As can be seen, these methods of locating after removing far-shot and recognition are based on the advantages of the RGB-D camera, which can obtain depth and color information synchronously. However, these methods still rely on traditional simple color information to recognize fruits and do not take advantage of the depth information or avoid the deficiencies of conventional machine vision based on CCD cameras for fruit recognition and location.

Nguyen et al. [14] used Asus Xtion to obtain data points of an apple tree. After removing the far-shot redundant information by the distance filter and filtering out the green leaf background by the red-green (R-G) color filter, the area of fruits can be obtained based on point-cloud clustering. Mai et al. [15] combined RGB-D information of an apple tree collected by Kinect V2 to reconstruct a color 3D model of the tree. They separated fruit and background based on the color threshold of R-G, removed the noise points based on an outlier filter of point clouds and extracted the 3D shape of each fruit point cloud by mapping the color-depth points. Lehnert et al. [16] reconstructed the 3D shape by fusing sweet peppers’ color and depth information and separating the red sweet pepper by the naive Bayes classifier in rotated HSI color space and obtained the 3D object fruit by Euclidean clustering. To recognize a single fruit, Tao et al. [17] separated the point clouds by color difference and depth data, extracted the color in the HSI and RGB color space and extracted the 3D geometric features by a quick point feature histogram descriptor based on depth-point cloud data. Qiu et al. [18] proposed a strategy to recognize fruit by selecting foreground with a depth threshold and operating green enhancement with a color threshold, which was based on RGB-D images of tomato plants obtained by Kinect V1. Chen et al. [19] used Asus Xtion at the head to get RGB-D information, removing the green leaf background by HSI thresholding and detecting clusters of tomatoes by depth-point cloud clustering, which can guide the arm to reach the close field of the tomato, and used the in-hand Prime Sense Carmine to obtain depth information to distinguish and locate the on-string fruit with the characteristics of a sphere. The above studies processed images with color differences between the fruit and the background and recognized the object fruit by judging the remaining points of clustering or geometric characteristics with depth information. This method makes the best of depth information in the process of fruit recognition.

Moreover, Choi et al. [20] used Kinect V2 to synchronize the RGB, near-infrared (NIR) and depth images of on-tree green citrus. They applied a 2D Hough transform algorithm to RGB and NIR images and Choi’s circle estimation algorithm for the depth image to search the object fruit, then detected the fruit from the background with the AlexNet classification model. The results show that the success rate of fruit detection based on depth was the lowest. Yasukawa et al. [21] provided a method of distinguishing a ripe red tomato from the background with RGB-HSV conversion based on color information and matching the gradient direction of infrared reflection intensity data obtained by the depth sensor to recognize and locate the fruit, which was based on the color information obtained by Kinect V2. The above methods made use of the infrared reflection intensity of the RGB-D sensor.

All of the studies effectively promoted new research on fruit recognition and localization based on the RGB-D sensor. According to the research, Kinect, Xtion and other cameras can obtain multiple information only in the range of 500–800 mm, which cannot be used in close-shot detection. Therefore, we have the following problems:

(1) In the range of a far-shot field of view, the defects of too many objects and complex redundant information lead to much interference, and a large amount of computation will influence the success rate and real-time application [18]. Limited by the resolution and accuracy of consumer RGB-D sensors, far-shot detection cannot obtain a clear depth image of the fruit and will make significant positioning errors [12].
(2) The consumer RGB-D sensors acquire the depth and NIR intensity data actively with a low-power NIR emitter-receiver and capture the RGB image with CCD detection passively. Both are challenging to adapt to outdoor natural light conditions effectively, and thus, the existing studies are mainly based on stable indoor light conditions [19] or the enclosed light environment [14].
(3) According to many studies, the far-shot location of the “eye-in-hand” cannot meet the requirements of picking accuracy. The open-loop control leads to a decline in picking performance, affecting the picking cycle and success rate [22,23]. As a result, the hand-eye coordination of the eye-in-hand [24,25,26] and image-based “look and move” [23,27] has become a trend of picking robots. However, Kinect, Xtion and other cameras cannot be applied to close-shot detection due to the limitations in detection range and size.

To break through the limitations of RGB-D sensors in robotic picking applications, Lehnert et al. [16] and Chen et al. [19] equipped RealSense and Carmine on robots’ hands, respectively. They only discuss the feature extraction of fruit and its corresponding peduncle in the close shot and do not solve the problem of how to detect fruit in a close-shot canopy background. They do not offer an expanded discussion on the application of in-hand RGB-D cameras, close-shot recognition and location and the servo features.

In this paper, the close-shot detection of citrus based on a new RGB-D camera, RealSense, is discussed.

## 2. Close-Shot Depth Information Collection with RealSense

### 2.1. RealSense Camera

**Figure 1:**
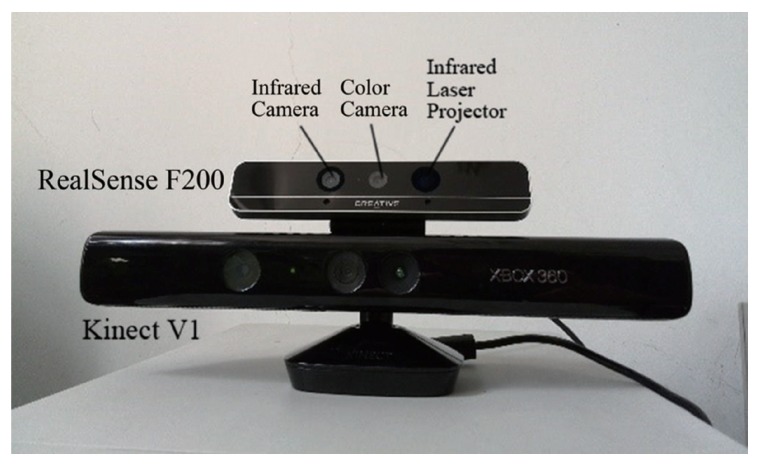
RealSense F200 vs. Kinect V1.

The RealSense camera is an integrated RGB-D somatosensory device launched by Intel in 2014. It is similar to other somatosensory cameras that use time-of-flight (TOF) theory to obtain depth information, as shown in Figure 1. As seen in Figure 1 and Table 1, the RealSense camera has advantages in size-detecting range accuracy, and so on, so it has been widely integrated into flat-panel screens, PCs and other equipment, as well as applied in unmanned aerial vehicles, intelligent robots, and so on.

**Figure 2:**
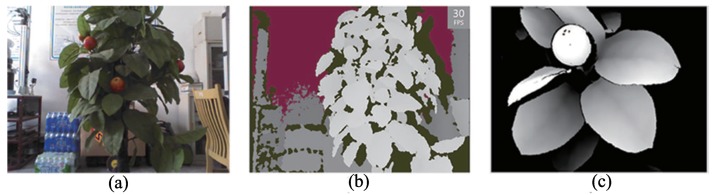
Comparison of depth images between Kinect and RealSense: (**a**) the real scene; (**b**) depth image captured with Kinect (distance: 0.9 m); (**c**) depth image captured with RealSense (distance: 0.3 m).

Compared with widely-used RGB-D cameras such as Kinect, RealSense can achieve close-shot detections of a 200-mm distance and obtain a high-definition 3D depth data stream with a resolution of 640×480. The frame rate is 60 frames per second to obtain the depth-point cloud data of the target fruit and leaf, which is more intensive than the data obtained by Kinect at a greater distance, as shown in Figure 2. Those features show that the 3D depth advantage of somatosensory equipment can be effectively used in fruit recognition.

### 2.2. Acquisition of Depth Information with RealSense

**Figure 3:**
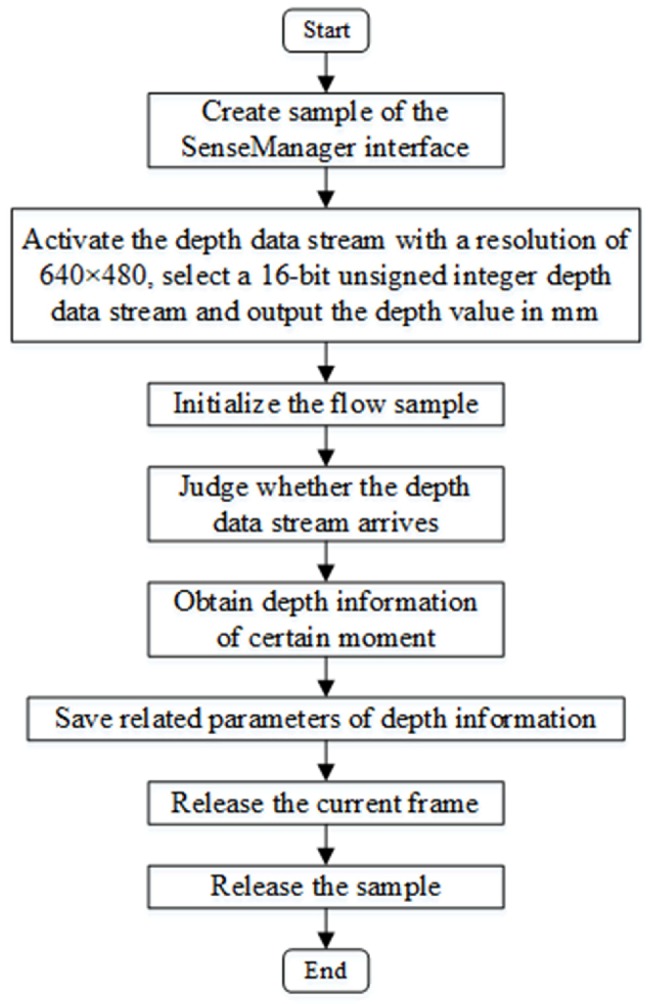
The algorithm flow of the acquisition of depth information with RealSense.

**Figure 4:**
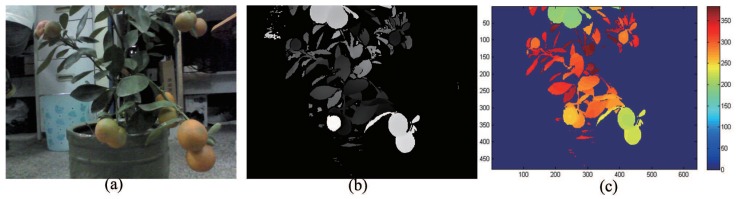
A depth-point cloud of a kumquat plant captured by RealSense F200: (**a**) color image; (**b**) depth image; (**c**) rendered depth image.

The real code of the depth data is completed on a computer installed with Windows 8.1 and the Microsoft Visual Studio 2013 software platform. Firstly, create an ImageData and ImageInfo type object, and call the EnableStream function to acquire the depth data stream. Secondly, select the ushort type pointer dpixels to point to the first depth cache address. Next, use integer dpitch to determine the widths of a frame of data. Finally, obtain the depth value dpixels [y× dpitch + *x*] corresponding to the midpoint p(x,y) through a circular offset and output it to the text document. There are 640×480 data points in each frame. The algorithm flow is shown in Figure 3. The original color image and original depth images are shown in Figure 4.

Figure 4 shows a depth image reconstructed and rendered with the original depth-point cloud data. Before the recognition experiments with RealSense, an experiment to calibrate the real recognition range of RealSense was carried out. As a result, RealSense can obtain steady depth data at a distance of 160 mm, so it can be used in close-shot detection. When the detection distance is in the close-shot range of 160–700 mm, it is possible to obtain a clear and stable depth image of fruit and leaf in the field of view, to make the data noise and jump error small and to leave out the preprocessing of filtering denoising. These advantages can be used to detect fruit mainly based on depth data.

## 3. Method and Strategy of Close-Shot Fruit Recognition

### 3.1. Depth-Sphere Cutting Theory

**Figure 5:**
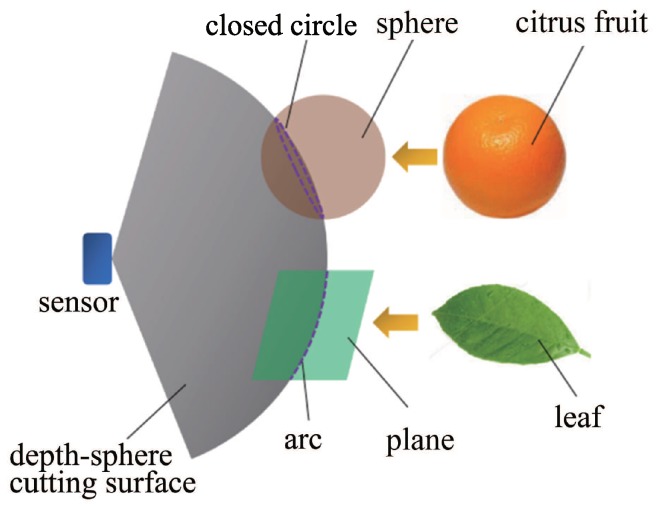
Depth-sphere cutting theory.

Considering that the three-dimensional geometric characteristics of the fruit and leaf targets of citrus are a sphere and a plane, the intersection curve of the closed circle and arc can be obtained respectively when the spherical surface intersects with two types of geometry. The fruit and leaf can be distinguished by the different shapes of the intersection curve, as shown in Figure 5.

The 3D depth-point cloud data obtained by RealSense is the distance between the detected point and the focal plane of the sensor. Without any conversion computation, the intersection curves of the objects in the field of view can be obtained by cutting with a sphere, which takes the center of the sensor’s focal plane as the center and the depth *D* as the radius. This way to distinguish fruit and leaf is quick and easy, with minimal computation.

### 3.2. Fundamental Parameters of the Applications of the Depth-Sphere Cutting Theory

#### 3.2.1. Closest Point of the Point Cloud

**Figure 6:**
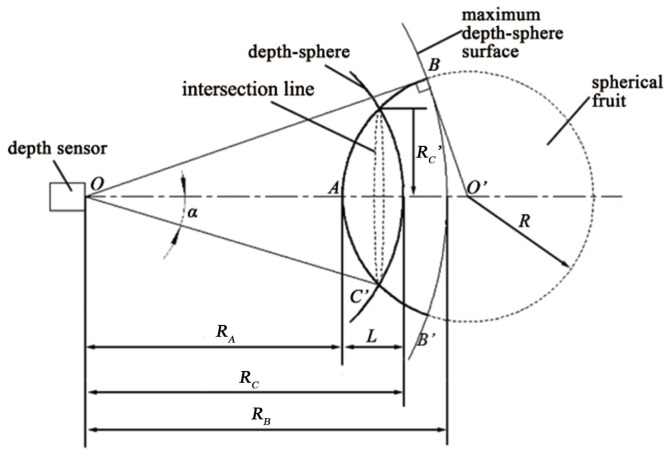
Schematic plan of a spherical entity by depth-sphere cutting.

The depth-sphere cutting method to recognize fruit is dependent on the different shapes of the intersection curves. In practical applications, the radius of the depth-sphere (called *R*), which is determined by the point-cloud aggregation of the objects, is the key for it to be cut by the depth-sphere for an effective intersection curve. As shown in Figure 6, the depth-sphere radius *R* is related to the closest point *A* of the point cloud and the cutting depth *L*. For independent geometry, the closest point *A* of the point cloud can be obtained directly. *A* should meet the formula:

(1) A=P(min(D),θ,ϕ)

where *P* is the spatial coordinate point in the coordinate system, *D* is the depth of the point, θ is the inner deflection angle of the horizontal surface and ϕ is the inner deflection angle of the vertical surface.

#### 3.2.2. Cutting Depth

The adhesion of fruit and leaf results in a complex geometric structure. RealSense has sufficient resolution and depth accuracy. Therefore, the intersection curves of target fruits or leaves depend on the depth of the depth-sphere cutting the target. As shown in Figure 6, the small intersection circle for the small cutting depth and the actual shape of the citrus and surface roughness of the peel make it difficult to obtain a satisfactory intersection circle. Since the data for that part cannot be detected by the sensor, the cutting depth will be limited by the depth of the farthest point. To increase the amount of depth data, the cutting depth *L* should be increased.

As shown in Figure 6, the sphere cutting depth should be:

(2) $$L=R_{C}-R_{A}$$

where *L* is cutting depth, which is the depth of cutting into the fruit surface by the depth-sphere, $R_{C}$ is the radius of the depth-sphere and $R_{A}$ is the depth of the closest point of the fruit point cloud, all in mm.

According to the geometric relationship of ideal spherical fruit shown in Figure 5, Formula (2) can be rewritten as:

(3) $$L=R_{C}-\sqrt{R_{C}^{2}-{R_{C}}^{\prime 2}}+R-\sqrt{R^{2}-{R_{C}}^{\prime 2}}$$

where ${R_{C}}^{\prime }$ is the radius of the circular intersection curve and *R* is the radius of the spherical fruit, both in mm.

The amount of depth data, which is obtained by cutting the citrus fruit with the depth-sphere, is decided by the resolution, the depth of field and the size of the intersection circle:

(4) $$N_{1}=\frac{2k_{a}\pi {R_{c}}^{\prime }}{R_{c}}{\left(\frac{m}{\theta_{0}},\frac{n}{\varphi_{0}}\right)}_{min}$$

where $N_{1}$ is the number of depth points for the intersection circle of citrus fruit, $\theta_{0}$ is the detection angle of the depth sensor in the horizontal direction, $\varphi_{0}$ is the angle in the vertical direction, *m* is the resolution of the depth sensor in the horizontal direction, *n* is the resolution in the vertical direction and $k_{a}$ is the factor of safety, which is decided on the polar radius, equatorial radius, shape error and surface roughness of the fruit. In the equation, $k_{a}$ is always less than one.

Combining Formulas (2)–(4), we get Formula (5):

(5) $$N_{1}=\frac{2k_{a}\pi }{L+R_{A}}\left(\frac{m}{\theta_{0}}∼\frac{n}{\varphi_{0}}\right)\sqrt{R^{2}-{\left(\frac{2R^{2}+2\left(R-L\right)R_{A}-L^{2}}{2(R_{A}+R)}\right)}^{2}}$$

The amount of depth data $N_{1}$ of the depth-sphere cutting is crucial to the curve of the intersection circle.

### 3.3. The Strategy of Close-Shot Fruit Recognition

#### 3.3.1. Special Working Conditions in the Complex On-Branch Environment

In the complex on-branch environment, the adhesion of fruits and leaves leads the point cloud to connect as a whole. Moreover, for the interlacing of fruits, leaves and branches lead to the fruits and leaves being connected, interlacing the branches. Compared with isolated, individual fruits and leaves with different geometric characteristics, connected objects make it impossible to use the method of depth-sphere cutting based on the closest point.

According to the working conditions of the on-branch environment and the requirement of applying the method of depth-sphere cutting, it is necessary to realize the discretization of the point cloud aggregation in this complex environment, that is to obtain the point cloud aggregation of the unconnected objects to apply the method of depth-sphere cutting.

#### 3.3.2. Point-Cloud Clustering and Aggregate Discretization

In the complex on-branch environment, the point cloud of the branch with the characteristics of a fine column in depth detection is a discrete or continuous line. The point cloud of the branch should be filtered out with point-cloud clustering to obtain some unconnected areas of point-cloud aggregation containing fruit and leaf objects.

In the area of point-cloud aggregation, the fruits and leaves can be isolated and adherent with each other. With this complex condition, the area should be divided into isolated and adherent with the point-cloud number threshold $N_{0}$. The corresponding feature strategy was used to identify the fruits in different areas.

#### 3.3.3. Characteristics and Process of Adherent Aggregate

In the canopy, there are occlusion phenomena in the field of view of the sensor, which severely affect the success rate of fruit recognition. However, the depth sensor can obtain the depth-point cloud of a single object with the method of depth-sphere cutting when the fruits and leaves are not adherent with each other in an anterior-posterior direction of the 3D space.

**Figure 7:**
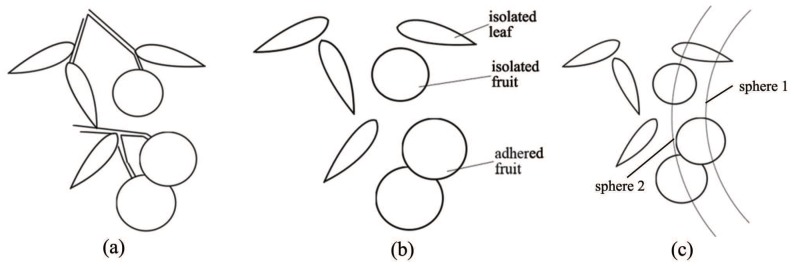
Schematic diagram of the information detection of on-branch citrus: (**a**) canopy; (**b**) clustering; (**c**) multiple depth-sphere cutting.

It is difficult to apply the method of depth-sphere cutting to the intricate geometric characteristics of point-cloud aggregation, which is caused by fruits adhering to leaves or other fruits. As shown in Figure 7, the isolated intersection curve of fruits and leaves can be obtained by cutting the adhering point cloud with the depth-sphere into different sizes. For this purpose, the cutting depth should be successively increased from the closest point to gain the characteristic intersection curve of the fruits and leaves. To complete the recognition of adhered fruits, further merge the same object by the special position relations of the characteristic intersection curve.

#### 3.3.4. Strategy and Process of Close-Shot Recognition of On-Branch Fruit

**Figure 8:**
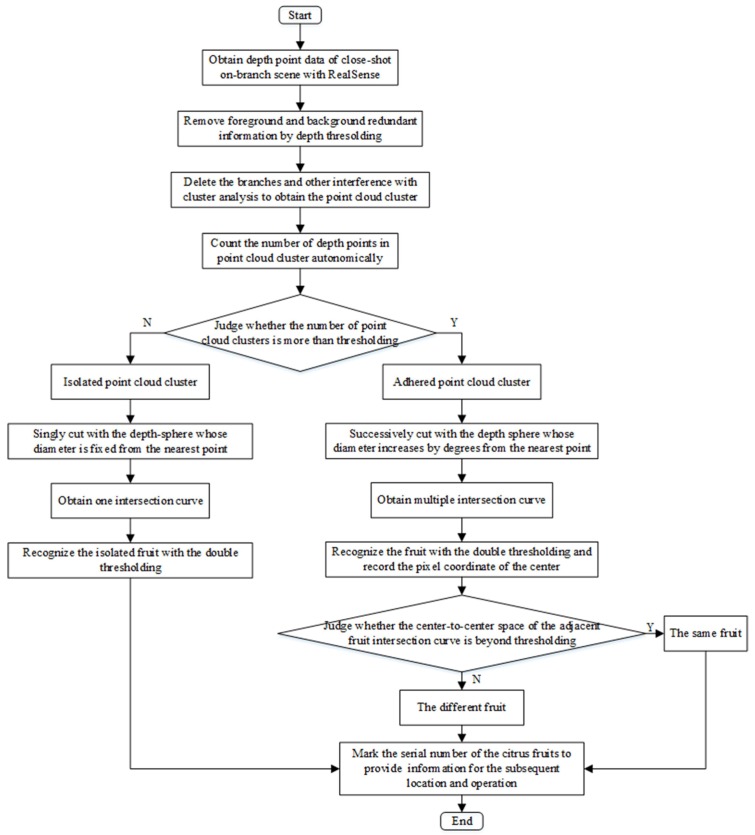
Flowchart of fruit recognition in the complex on-branch environment.

According to the above analysis, the close-shot recognition process of on-branch fruit based on the depth-sphere intersection curve theory is shown in Figure 8. Firstly, determine the close-shot detection range of 160–700 mm by depth thresholding. Secondly, divide the isolated and adhering areas of on-branch citrus fruits and leaves according to the difference in the number of point clouds in each point-cloud aggregation region after being processed. Lastly, detect the fruit by a single and successive depth-sphere cutting algorithm.

In the case of fruits and leaves touching each other, only a partial intersection curve can be obtained with the depth-sphere cutting method. For this reason, to distinguish fruit and leaf objects, both the eccentricity and number of pixels should be treated as the double thresholding [30]:

(6) $$\left\{\begin{array}{c} E\left\{C\left(D_{C}\right)\right\}\leq E_{0}\\ A_{N}\left\{C\left(D_{C}\right)\right\}\geq A(N_{0}) \end{array}\right.$$

where $C(D_{C})$ is the object intersection curve by the method of depth-sphere cutting and *E* is the eccentricity, whose second central moment is the same as each intersection curve area in a specific standard, which shows the curvature of the connected domain. The eccentricity *E* is more significant than the perfect circle’s, which is zero, is smaller than the line’s, which is one, and is used to filter out the leaf objects effectively. $E_{0}$ is the eccentricity threshold of the intersection curve connected domain. $A_{N}$ is the number of depth pixel points in each connected domain of the intersection curve. Considering the differences in size of the intersection curve of citrus fruit, leaf and branch, the number of pixels can be used to filter out the branches and leaves effectively. $A_{N0}$ is the pixel threshold of the connected intersection curve domain.

## 4. Experiments

### 4.1. Materials

**Figure 9:**
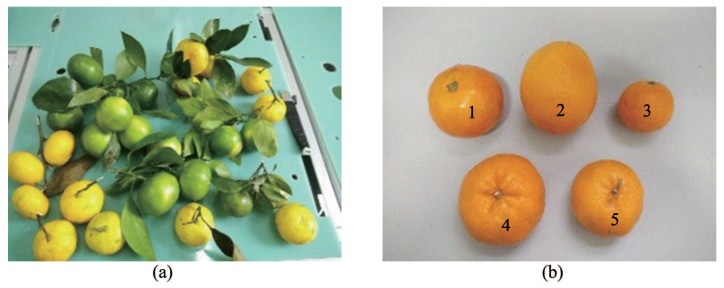
Six varieties of citrus fruits: (**a**) Jiangxinzhou tangerine; (**b**) 1. Gannan navel orange; 2. Egyptian orange; 3. Yunnan crystal sugar orange; 4. Sichuan ugly orange; 5. Yongchun ponkan.

The experiments were conducted at the Agricultural Robot Laboratory of Jiangsu University in September 2016. One hundred fresh ripe tangerines were picked randomly in Jiangxinzhou orchard in Zhenjiang, while ten each of fresh Gannan navel oranges, Egyptian oranges, Yunnan sugar oranges, Sichuan ugly oranges and Yongchun ponkans were purchased randomly as samples. The fruits are shown in Figure 9.

The polar diameter and equatorial diameter of each citrus fruit were measured with an electronic digital display vernier caliper (precision 0.1 mm), and their characteristic coefficients were calculated. All the results are shown in Table 2.

The cutting depth, crucial to obtaining the ideal depth-sphere transversal of the fruit, should be determined by the size of the citrus fruit in the experiment. According to Formula (5), the depth points of the double thresholding of the incomplete intersection curve (≥50%) can be determined. The radius of the fruit and the depth of field of close-shot detection can be determined by statistics. The safety factor $k_{a}$ is 0.8, and the values of minimum cutting depth *L* (integer-valued) of the different citrus fruits are shown in Table 2.

### 4.2. Methods

#### 4.2.1. Recognition of Isolated Objects without Occlusion

**Figure 10:**
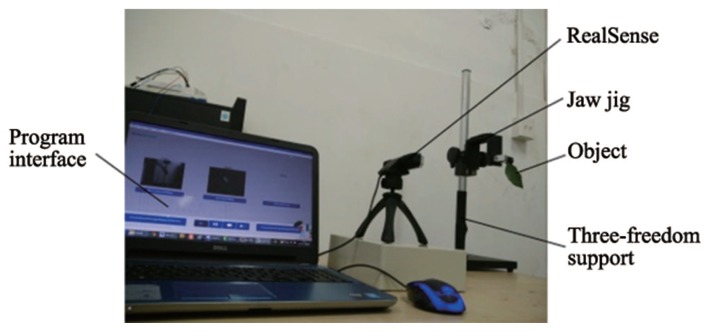
Experiment device and scene.

**Figure 11:**
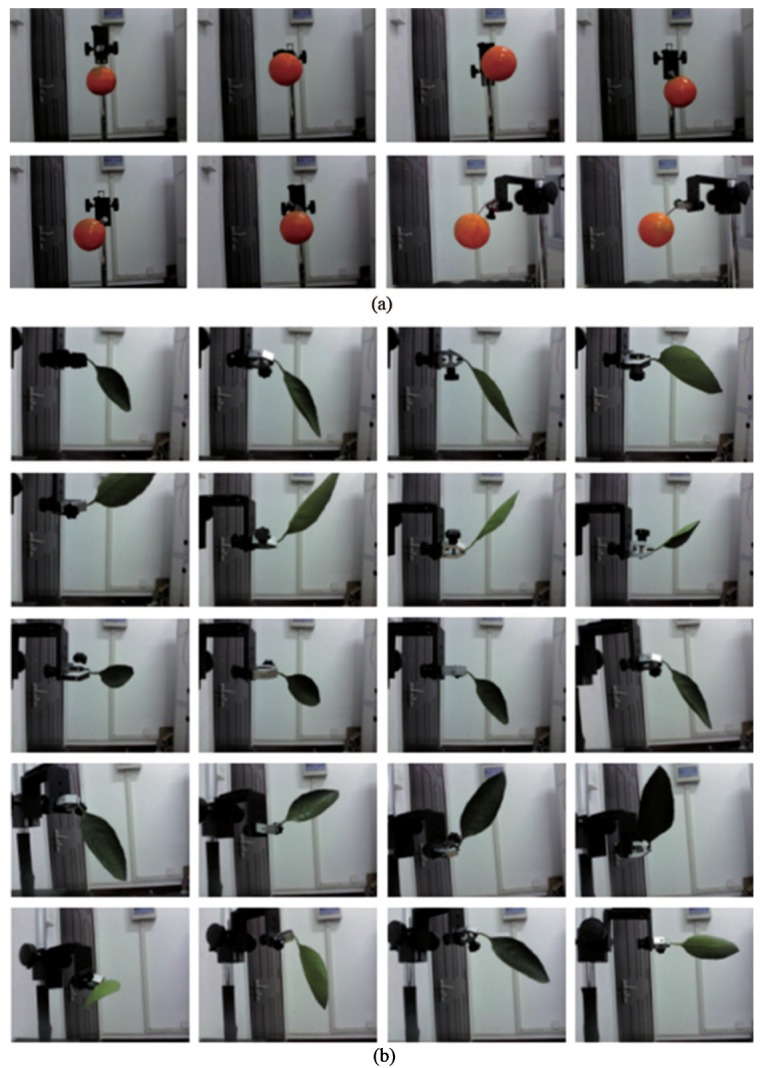
Position-postures of the isolated objects: (**a**) eight kinds of fruit position-postures; (**b**) twenty kinds of leaf position-postures.

The equipment is shown in Figure 10. There are 8 pipless tangerines, and 6 leaves are taken as samples for the experiment. The RealSense F200 was fixed on a miniature tripod whose plane was perpendicular to the desktop. In the close-shot range of 160–700 mm, to imitate the actual growth state of the on-branch environment as much as possible, the position-posture of the clamped object should be adjusted by the three-freedom support and a jaw jig. There were 8 random different position-postures for each fruit, and the depth information of the 64 total positions was collected. For that, the leaf morphologies on the tree were varied. One hundred twenty pieces of leaf scene depth information were collected with 20 position-postures for each leaf. The position-postures of isolated fruit and isolated leaf are shown in Figure 11. In the experiments, the used computer model is DELL Inspiron 15R-5537, which is equipped with Intel(R) Core (TM)i7-4500U processor, and it uses the 64-bit Windows 8.1 system. Then, the depth information of the fruit, leaf and branch were distinguished with the feature extraction strategy of fruits, using MATLAB.

#### 4.2.2. Recognition of Citrus Fruits from Different Fruit-Leaf Collocations

**Figure 12:**
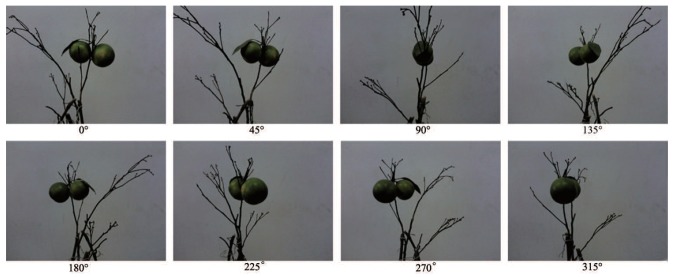
Experiment with two fruits and one leaf.

The fruits and leaves of fresh Jiangxinzhou tangerines are taken as experiment targets. The diverse number of fruits and leaves are collocated to present different position-postures randomly. There are 5 sets of experiments with one fruit and one leaf, 5 sets with one fruit and two leaves, 5 sets with one fruit and three or four leaves, 4 sets with two fruits and one leaf, 5 sets with two fruits and two leaves and 6 sets with two fruits and three or four leaves. For each set, the pot should be rotated horizontally every 45${}^{\circ }$ in the same place. The experiment of two fruits and one leaf is taken as an example, shown in Figure 12. The amount of experiments is shown in Table 3.

#### 4.2.3. Recognition of Various Varieties of Citrus Fruits with Occlusive and Adhering Leaves

**Figure 13:**
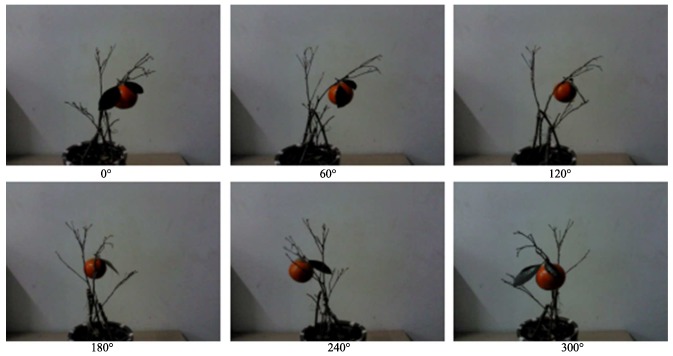
A set of citrus experiment position-postures.

In the case of a single fruit with 2 leaves, 10 each of Jiangxinzhou tangerines, Gannan navel oranges, Egyptian oranges, Yunnan crystal sugar oranges, Sichuan ugly oranges and Yongchun ponkans were randomly hung in potted citrus branches to show different position-postures. The pot should be rotated horizontally every 60${}^{\circ }$ in the same place as shown in Figure 13 to collect the horizontal depth-point cloud in the close-shot range for each position-posture. Then, the feature extraction algorithm is used to identify the adhering fruit.

**Figure 14:**
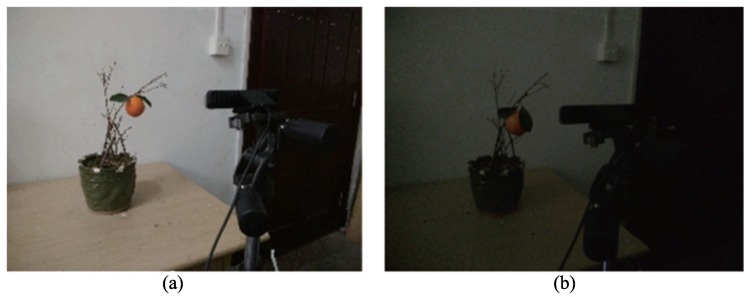
Experimental scene of citrus fruit and leaf: (**a**) light environment; (**b**) dark environment.

Light conditions are a vital factor in fruit recognition technology. To test the efficiency of RealSense close-shot fruit recognition in the dark and compare it with efficacy in the light, the indoor light environment and dark environment were set for each experiment, as shown in Figure 14. The experiment in the light was carried out with an incandescent lamp from 1–4 p.m., while the experiment in the dark was carried out without light from 7–10 p.m.

#### 4.2.4. Recognition of On-Branch Citrus Fruit

**Figure 15:**
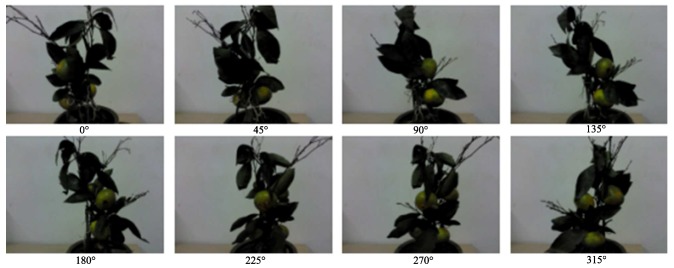
A set of complex fruits and leaves experiment position-postures on a branch.

Each experiment was set in 10 random on-branch collocations of the fruits and leaves. The collocation means that there are three or four Jiangxinzhou tangerine fruits chosen to be set as isolated and adherent to other fruits and leaves. For each position-posture, the pot should be rotated horizontally every 45${}^{\circ }$ in the same place. The depth information on 80 complex scenes was collected in the horizontal directions with RealSense in close shot, and the complex environment of the fruit feature extraction strategy was used to judge. A set of complex fruits and leaves experimental position-postures on the branch is taken as the example, which are shown in Figure 15.

### 4.3. Result

#### 4.3.1. Recognition of Isolated Objects

**Figure 16:**
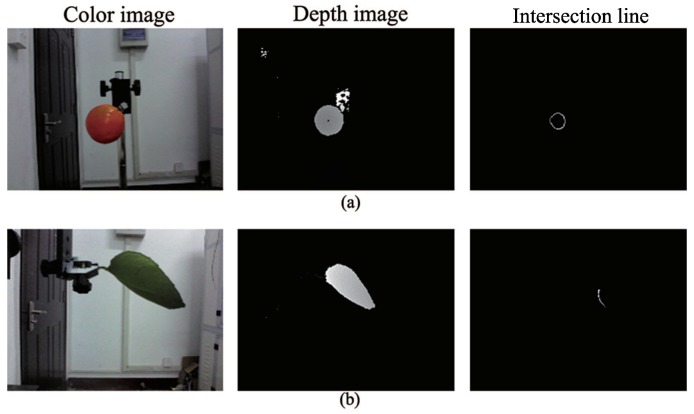
Objects with corresponding intersection curves in an isolated area: (**a**) isolated fruit; (**b**) isolated leaf.

The results of isolated fruit and leaf with the depth-sphere intersection curve are shown in Figure 16. There were 63 intersection curves successfully discriminated as fruit in the 64 fruit scenes, and there were 117 intersection curves successfully ruled out as fruit in 120 blade scenes. The total recognition rate of isolated objects was 97.8%, which is shown in Table 4.

#### 4.3.2. Recognition of Multiple Position-Postures of Different Fruit-Leave Collocations

**Figure 17:**
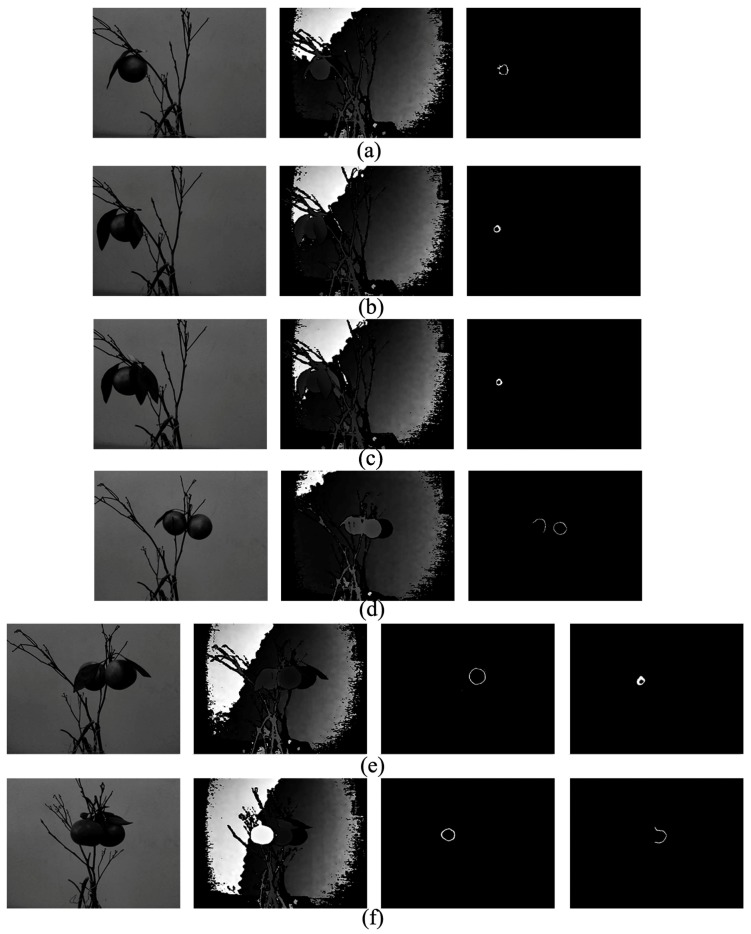
Successful fruit intersection curves of the experiment of different fruit-leave collocations: (**a**) one fruit and one leaf; (**b**) one fruit and two leaves; (**c**) one fruit and multiple leaves; (**d**) two fruits and one leaf; (**e**) two fruits and two leaves; (**f**) two fruits and multiple leaves.

The results of the experiment of different fruit-leave collocations are shown in Figure 17. The total recognition rates of each collocation are shown in Table 5. In the experiment of one fruit and one leaf, the total recognition rate is 82.5%. In total, with the increase of the number of fruits and leaves, the adhesion and occlusion phenomena are more severe, and the success rate of recognition decreases. However, it can be found in Table 5 that the success rate of the experiment of one fruit and multiple leaves is lower than two fruits and multiple leaves. The results are related to the experimental treatment. In the experiment, there were one or two fruits collocated with multiple leaves (three or four leaves), so the occlusion of the experiment of one fruit is more severe than that of two fruits.

#### 4.3.3. Recognition of Multiple Citrus Fruits

**Figure 18:**
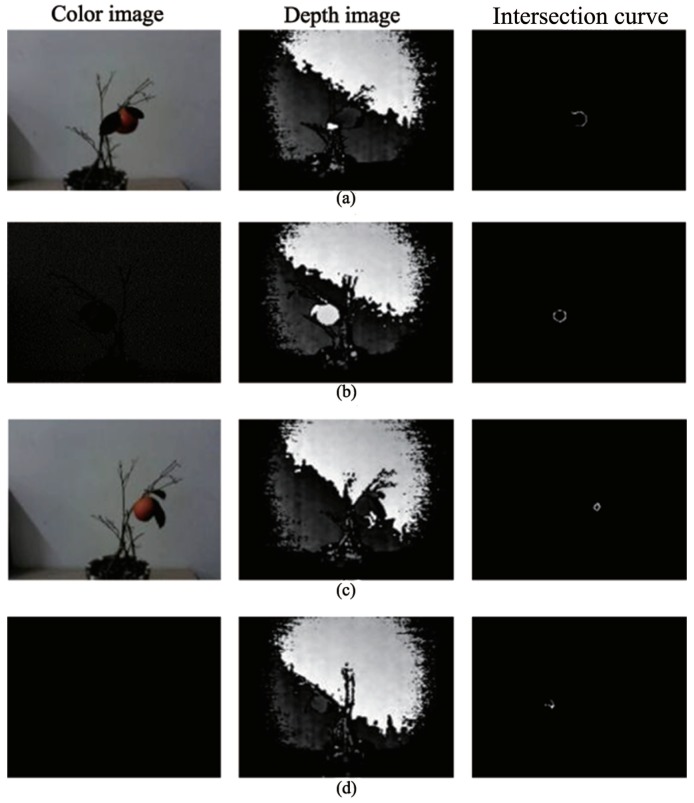
Typical oranges and their corresponding intersection curves: (**a**) light environment (Gannan navel orange); (**b**) dark environment (Gannan navel orange); (**c**) light environment (Sichuan ugly orange); (**d**) dark environment (Sichuan ugly orange).

The fruit intersection curves of Gannan navel orange and Sichuan ugly orange that depend on cutting depth (see Table 2) are shown in Figure 18.

**Figure 19:**
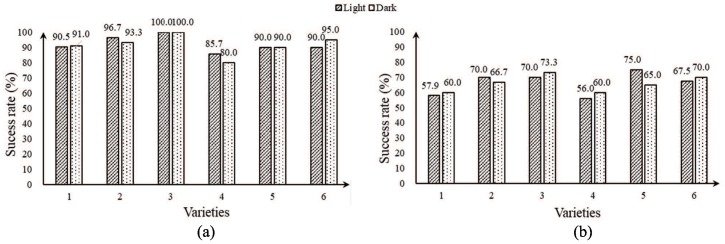
Recognition results of six types of citrus: (**a**) little occlusion and adhesion; (**b**) serious occlusion and adhesion. 1. Jiangxinzhou tangerine; 2. Gannan navel orange; 3. Egyptian orange; 4. Yunnan sugar orange; 5. Sichuan ugly orange; 6. Yongchun ponkan. Comments: Little occlusion and adhesion mean that the fruit can be detected at >50% of the surface and few parts of the fruit and leaf have adhesion. Serious occlusion and adhesion mean that the fruit can be detected at ≤50% of the surface and the main body parts of the fruit and leaf have obvious adhesion.

The results of comparing six kinds of on-branch citrus fruits with little occlusion and severe occlusion are shown in Figure 19. The results show that little occlusion, which led to significant depth-point clouds of fruit, and little adhesion, which could be cut easily by depth-sphere cutting to obtain a single intersection curve, brought the rate of fruit recognition up to 80–100%. Serious occlusion, which made the proportion of detected fruit surface too small, and severe adhesion, which made it difficult to divide the point cloud clustering by the depth-sphere, brought the rate of fruit recognition down to 56–75%.

Comparing the rate of recognition in the light and the dark, we can see that there is no obvious difference in the detection rate between the light and the dark. However, a clear and stable depth image can be obtained for the active light source mode of depth detection and the intensity of the close-shot light source and received reflected light. Therefore, the combination of the RealSense hardware advantages and close-shot detection algorithms makes possible all-weather detection and robot harvesting.

Furthermore, the detection rates of different citrus fruits were different. The detection rate of the Egyptian orange was the highest. With little occlusion and adhesion, the success rates of detecting Gannan navel orange, Sichuan ugly orange, Yongchun ponkan and Jiangxinzhou tangerine were all above 90%; the latter was smaller than the former, and Yunnan crystal sugar orange was the lowest. With serious occlusion and adhesion, the success rates of detecting Sichuan ugly orange, Gannan navel orange and Yongchun ponkan reached 65–75%, but that of Jiangxinzhou tangerines and Yunnan crystal sugar oranges were both below 60%.

#### 4.3.4. Recognition of Complex On-Branch Citrus Fruits

**Figure 20:**
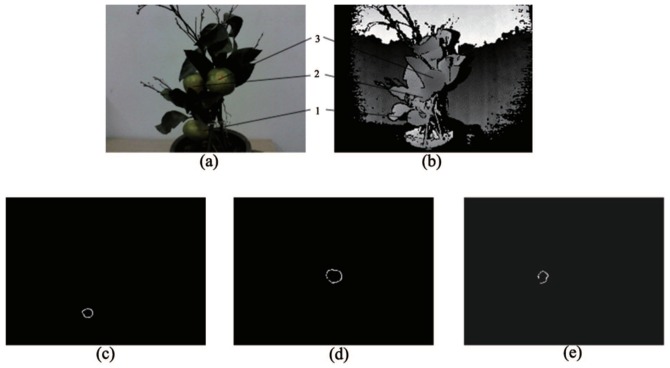
Successful fruit intersection curves in a complex environment: (**a**) complex on-branch environment; (**b**) depth image; (**c**) intersection curve of Fruit 1; (**d**) intersection curve of Fruit 3; (**e**) intersection curve of Fruit 2.

The fruit intersection curves in the complex on-branch environment containing isolated fruits and leaves and adhesion of fruit-fruit and fruit-leaf are shown in Figure 20. In the complex on-branch environments, the process shown in Figure 8 can be realized quickly to make the success rate of fruit recognition 63.8%. The success rate is a combined result of the clustering and classification detection performance of isolated and adhering fruits under the condition that the relationship between RealSense and the on-branch environment is fixed. In applications, changing the position of RealSense not only enhances the issue of obtaining depth data of fruits with serious occlusion, but also successfully separates and detects adhered fruits, to greatly improve the success rate of fruit detection.

## 5. Conclusions

### 5.1. Reliability of RealSense Close-Shot Recognition

(1) Influence of leaf blade morphology

**Figure 21:**
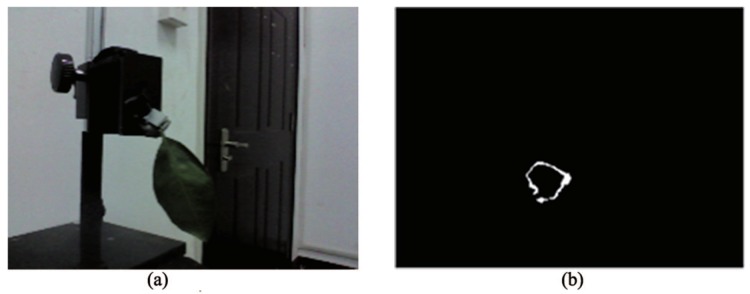
Color image of curly leaf and corresponding depth-sphere intersection curve: (**a**) color image; (**b**) intersection curve.

As shown in Figure 21, when the blades are very curly and cut by the depth-sphere at a coincident angle, it is possible to obtain the apparent curved intersection arc of the object leaf blades so that the leaves are misjudged as fruit. However, in the natural environment with a canopy, misrecognition caused by this situation is rare. The rate of misrecognizing the isolated blade is only 2.5%. However, in the on-branch environments, the rate of misrecognition is 3.3% and 5.3% for little and severe adhesion, respectively.

To avoid misrecognition of extreme position-postures, the depth information can be cut after adjusting the position-posture of the leaf, changing the angle of view and the center of the sensor (the center of the depth-sphere).

(2) Influence of fruit size and shape 

For isolated fruit, the success rate of detection based on the depth-sphere intersection curve was over 98%. However, in the complex on-branch environment, there will be false negatives of fruit objects, and the different sizes and shapes of different fruits influence the success rate of detection. Meanwhile, compared with the circularity of citrus fruit, the size of the fruit has a more obvious influence on the success rate of detection. As shown in Figure 19, the recognition rate of Egyptian oranges, whose size is most extensive, with average polar diameter and equatorial diameters of 86.6 mm and 77.9 mm, respectively, reached 100% and 70% with little adhesion and serious adhesion, respectively. For Yunnan crystal sugar orange, which is approximated by a circle with an average polar diameter and equatorial diameter of only 49.9 mm and 54.3 mm, respectively, the success rate of detection was the lowest.

The reason that different cutting depths were chosen for the different varieties of citrus in the same depth of field is that sufficient numbers of point clouds of the depth-sphere intersection curve can be obtained. However, for fruit with a tremendous size, depth-sphere cutting with a small cutting depth can still be implemented effectively in an environment with occlusion and adhesion, to obtain arc curves that are independent and have a greater range. The high rate of fruit recognition can be ensured.

(3) Impact of the degree of adhesion 

For the recognition technology based on visible light, it is hard to separate information on fruits with occlusion to obtain a single outline of the fruit in the complex on-branch environment. For the detection method with depth information, if there is no adhesion among the fruits and leaves, the point-cloud clustering will be independent according to the different depth in the canopy. RealSense makes a further contribution to the clarity and legibility of individual point-cloud clustering in close-shot range.

Therefore, the method can be used to obtain an independent intersection curve of fruit and detect the fruit according to the double threshold of eccentricity, and pixel number, except the sensor, is unable to obtain enough depth data points of the fruit because of the serious occlusion. Besides, for the condition of little adhesion, in which there is a limited connection between the point cloud of the fruits and leaves, the method can be used to detect the fruit easily by changing the cutting depth. Operation of the “eye-in-hand” robot in the close-shot range can avoid the treatment of serious occlusion and adhesion to ensure reliable recognition and harvest.

However, in the case of serious adhesion, the rate of fruit recognition with this is low. To solve this problem, further research on fruit recognition under serious adhesion is essential.

(4) Effects of light conditions 

Light is a key factor in recognition of fruit, and the passive detection technology based on CCD is greatly restricted under natural light and low light. The near-infrared active detection of the depth sensor can achieve recognition without light, which enables the harvesting robot to do night-and-day work in the natural environment. Meanwhile, the consumer-grade depth sensors such as Kinect and front-facing RealSense can be easily manipulated outside for their small power. Thanks to the advantage of its minimum close-shot detection distance (160 mm), RealSense will obtain enough infrared light so that it can effectively overcome the natural light interference and achieve reliable close-shot detections of fruit, which was confirmed in a study on grape detection.

The existing study shows that the fruit recognition with this method has good results under a dark environment. However, the effect of this method under natural light conditions needs to be verified in further research.

### 5.2. Calculation of RealSense Close-Shot Recognition

To meet the requirements of the practical application of the harvesting robot, compared to object visualization and 3D modeling, real-time RealSense close-shot recognition and servo control have a great impact on performance and practical value. Therefore, computation of the positioning algorithm is crucial. The depth-sphere intersection curve algorithm of RealSense close-shot recognition has outstanding advantages, as follows:

(1) Close-shot detection for fewer targets 

**Figure 22:**
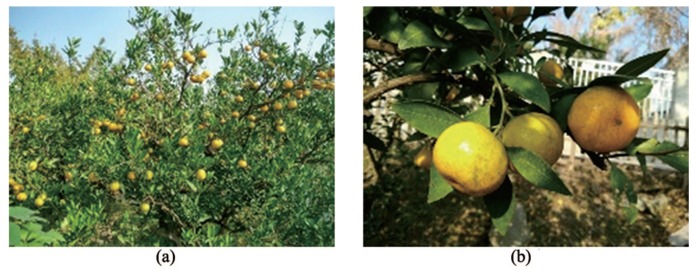
Comparison of the prospect and close-shot of citrus fruit: (**a**) far shot; (**b**) close shot.

In the far-shot field of view, there are some problems, such as too many objects, complex redundant information and the object being “farther, little, and blurry”, which make it difficult to recognize fruit and increase the amount of calculation. In the close-shot field of view, there are few objects and little redundant information, and the object is “close, large, and clear” (shown in Figure 22). RealSense can obtain high-precision depth data on a limited number of fruits and leaves in the close-shot field, among which the proportion of adequate data is large, making the surface spatial features of each citrus fruit and leaf object more prominent; which greatly reduces the difficulty of recognition, the amount of calculation and the number of errors.

For robotic harvesting, multiple objects in the far-shot canopy and rough detection of the complex environments cannot be directly used for feedback control. Compared with CCD, whose reliability and real-time performance of recognition and location are limited, and Kinect and Xtion, which cannot be used for their far detection range and big size, RealSense can be integrated with the end-effector of the harvesting robot based on its small size and close depth detection range of 160 mm. In addition, the fast depth detection of “large and clear” for close-shot objects meets the required precision of the robot hand-arm for locating and harvesting one-by-one, which makes RealSense the best choice for the real-time servo operation of harvesting robots.

(2) Rapid elimination of foreground and background 

Based on a close-shot detection range of 160–700 mm, redundant background, hole noise and unstable foreground can be eliminated with depth thresholding to quickly obtain the citrus point cloud, to avoid complex background interference of the remaining limited fruit and leaf objects. This can decrease the difficulty of recognition.

**Figure 23:**
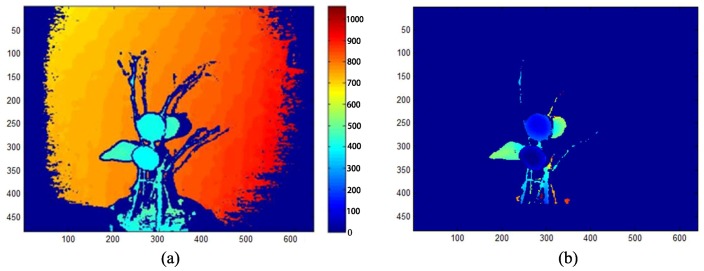
Elimination of foreground and background: (**a**) original depth image; (**b**) processed image.

We can determine by counting the point clouds of 100 on-branch citrus fruits in the close-shot detection range that the size of the point cloud can be decreased from 307,200 (640×480) to 6000–50,000 per frame. That is 2–16% of the original data, by rapid elimination of the background, which is shown in Figure 23. Thus, the calculation can be decreased to extract the fruit characteristics rapidly.

(3) Advantages of depth-sphere cutting algorithms 

For the existing method of fruit recognition based on depth information, segmentation of the fruit image is completed by 3D reconstruction with the depth-point cloud or the 2D image processing algorithm with the depth image. The method cannot take advantage of depth data to recognize the object fruit and then undergo calculation, which is still dependent on the traditional edge contour extraction algorithm and the complex analysis process of “obtain original depth data—visualization—calculate gray-scale value—extract the contour curve—identify fruit characteristics”.

By comparison, the depth-sphere cutting algorithm directly uses the sensor to obtain the original depth data and the spherical coordinates of the data. The depth-sphere cutting of each object can be directly realized without any data conversion operation to simplify high-precision computing, in order to ensure real-time detection and harvest.

### 5.3. Future Work

We aim to attempt a new technical route of on-branch fruit recognition. In this manuscript, the analysis is based on depth data directly instead of traditional image segmentation, and the new close-shot recognition method of depth-sphere cutting is promoted. Based on this method, we discover the features of isolated fruit and leaf and then increase the complexity of fruit-leaf collocation to realize the recognition of the on-branch fruits.

In this study, the recognition effect analysis for multiple fruit-leaf position-postures and collocations is completed in an indoor environment, and the integral recognition strategy/process and optimal parameters are obtained. However, in further work, it is necessary to verify the recognition effect in a natural field environment. Furthermore, the fruit recognition in the on-branch environment should be expanded to the on-tree environment. Finally, the research on fruit recognition under serious adhesion and occlusion is essential to further promote the recognition effect. The related research and its practical application in robotic picking are ongoing.

## Figures and Tables

**Table 1:** Comparison of the main official parameters of regular somatosensory cameras.

Reference	Type	Company	Resolution/PPI	Depth of Field (mm)	Frame Rate (fps)	Size (Length × Width × Height) (mm) (Including Stand)
[16,26]	RealSense F200	Intel	640×480	200–1200	60	136×25×55
[28,29]	Kinect V1	Microsoft	320×240	600–4000	30	282×63×70
[22]	Kinect V2	Microsoft	512×424	500–4500	30	250×85×65
[13]	Xtion	Asus	640×480	800–3500	30	180×36×50
[19]	Carmine	Primesense	640×480	350–3000	30	180×35×50

**Table 2:** Sphere section cut depth for each variety of citrus.

Varieties	Number of Samples	Polar Diameter $D_{p}$ (mm)	Equatorial Diameter $D_{e}$ (mm)	Shape Characteristic Coefficient $\rho =\frac{D_{p}}{D_{e}}$	Cutting Depth *L* (mm)
Jiangxinzhou tangerine	10	$43.2_{-9.9}^{+10.5}$	$55.0_{-5.9}^{+6.2}$	$0.785_{-0.107}^{+0.092}$	5
Yunnan sugar orange	10	$49.9_{-3.1}^{+2.2}$	$54.3_{-1.7}^{+0.8}$	$0.919_{-0.029}^{+0.027}$	6
Sichuan orange	10	$55.0_{-2.2}^{+4.3}$	$68.7_{-2.5}^{+2.9}$	$0.802_{-0.004}^{+0.026}$	4
Yongchun ponkan	10	$58.6_{-7.5}^{+7.9}$	$75.0_{-6.1}^{+3.8}$	$0.781_{-0.050}^{+0.063}$	4
Gannan navel orange	10	$66.9_{-2.8}^{+2.5}$	$71.4_{-3.0}^{+2.9}$	$0.937_{-0.003}^{+0.001}$	4
Egyptian orange	10	$86.6_{-2.1}^{+3.5}$	$77.9_{-1.5}^{+1.2}$	$1.111_{-0.005}^{+0.028}$	3

Notes: ρ is the characteristic coefficient of the fruit shape. 0.9≤ρ≤1, the fruit shape is nearly circular; ρ<0.9, the fruit shape is oblate; ρ>1, the fruit assumes an oval shape.

**Table 3:** Experiment plans for different fruit-leaf collocations.

	Fruits Number	Leaves Number	Sets	Number of Position-Postures	Experiment Amount
Experiment 1	1	1	5	8	40
Experiment 2	1	2	5	8	40
Experiment 3	1	3 or 4	5	8	40
Experiment 4	2	1	4	8	32
Experiment 5	2	2	5	8	40
Experiment 6	2	3 or 4	6	8	48

**Table 4:** Recognition rate of an isolated object.

Isolated Object	Sample Number	Number of Position-Posture	Experiment Amount	Rate of Misrecognition/%	Rate of Miss-Recognition/%	Success Rate/%
fruit	8	8	64	0	1.6	98.4
leaf	6	20	120	2.5	0	97.5
total						97.8

Notes: misrecognition is the error detection of fruit, which means recognizing leaves’ intersection curves as fruits by mistake; miss-recognition is lost detection of fruit, which means failing to recognize the fruits’ intersection curves.

**Table 5:** Recognition rate of different collocations.

	Number of Fruits	Success Amount	Number of Misrecognition	Number of Miss-Recognition	Success Rate/%
One fruit and one leaf	40	33	2	5	82.5
One fruit and two leaves	40	30	2	8	75.0
One fruit and multiple leaves	40	27	6	7	67.5
Two fruits and one leaf	64	51	0	10	79.7
Two fruits and two leaves	80	63	5	11	78.8
Two fruits and multiple leaves	96	70	5	15	72.9

## Abbreviations

The following abbreviations are used in this manuscript:

dpitch	pixel pointer of the depth image
dpixels	the depth of each pixel value
depth-sphere	sphere taking the origin of the RealSense coordinate as the center and the certain depth value as the radius of the depth-sphere
intersection curve	the surface curve obtained by cut with the depth-sphere
closest point	the point with the minimum depth value in the surface area of the detected fruit
cutting depth	the depth of cutting into a certain geometry by the depth-sphere, which is the difference between the depth of the closest point and the radius of the depth-sphere
adhesion	contact between fruits and leaves that makes the point cloud as a whole
occlusion	target fruit obscured (occluded) by other fruits or leaves in the field of view
intersection circle	intersection curve of the depth-sphere intersecting with a circular target to be a circle
misrecognition	error detection of fruit, which means recognizing leaves intersection curves as fruits by mistake
miss-recognition	lost detection of fruit, which means failing to recognize the fruits intersection curves
